# Circulating tumour cells for the prediction of the response to radiation therapy in prostate cancer

**DOI:** 10.1093/bjr/tqaf224

**Published:** 2025-09-13

**Authors:** Camille Landry, Daria Klusa, Denis Cochonneau, Stéphane Supiot, Dominique Heymann

**Affiliations:** Department of Radiation Oncology, Institut de Cancérologie de l’Ouest, Saint-Herblain, 44805, France; Tumour Heterogeneity and Precision Medicine” Laboratory, Institut de Cancérologie de l’Ouest, Saint-Herblain, 44805, France; Biological Sciences and Biotechnologies Unit, Nantes Université, CNRS, UMR 6286, US2B, Nantes, 44322, France; Tumour Heterogeneity and Precision Medicine” Laboratory, Institut de Cancérologie de l’Ouest, Saint-Herblain, 44805, France; Department of Radiation Oncology, Institut de Cancérologie de l’Ouest, Saint-Herblain, 44805, France; Tumour Heterogeneity and Precision Medicine” Laboratory, Institut de Cancérologie de l’Ouest, Saint-Herblain, 44805, France; Biological Sciences and Biotechnologies Unit, Nantes Université, CNRS, UMR 6286, US2B, Nantes, 44322, France; Tumour Heterogeneity and Precision Medicine” Laboratory, Institut de Cancérologie de l’Ouest, Saint-Herblain, 44805, France; Biological Sciences and Biotechnologies Unit, Nantes Université, CNRS, UMR 6286, US2B, Nantes, 44322, France; School of Medicine and Population Health, University of Sheffield, Medical School, Sheffield, S10 2RX, United Kingdom

**Keywords:** prostate cancer, circulating tumour cells, radiotherapy

## Abstract

Circulating tumour cells (CTCs) have emerged as a promising biomarker for assessing prognosis and predicting therapeutic efficacy in various cancers, including metastatic prostate cancer. However, predicting patient response to treatment, including radiation therapy (RT), remains a significant clinical challenge. This review explores the value of CTCs as prognostic markers in RT for prostate cancer, discussing their detection methods, biological significance, clinical relevance, and future implications.

## Introduction

Prostate cancer is one of the most common malignancies worldwide, particularly in men over the age of 50. It is the second leading cause of cancer-related death in men in the United States and other developed countries.^1^ The diagnosis and treatment of prostate cancer have evolved significantly over the years with advances in early detection and treatment modalities. Radiation therapy (RT) plays a central role in the management of both localized prostate cancer as primary or adjuvant treatment and oligometastatic cancer as a multimodal approach. Predicting how patients will respond to RT is a major challenge, as they do not all respond equally. Traditionally, the management of prostate cancer relies on prostate-specific antigen (PSA) level and imaging, such as prostate-specific membrane antigen positron emission tomography/computed tomography (PSMA PET/CT). However, they often fail to provide an early detection of treatment efficacy.^2^

Circulating tumour cells (CTCs) are epithelial or mesenchymal tumour cells shed from the primary tumour or metastases into body fluids (blood, urine, cerebrospinal fluid).^3^^,^^4^ Some of them can survive in the bloodstream, migrate by extravasation into the interstitial space, and form a distant tumour in a new microenvironment. They spread from the primary tumour either actively or passively during biopsy, surgery, and brachytherapy by dissemination and intravasation into blood vessels. In carcinomas, active dissemination occurs by the epithelial-to-mesenchymal transition, through factors like TGF-β, Wnt, or IL-6.^5^ Within the circulation, CTCs travel either alone, as homotypic clusters (CTC-CTC), or heterotypic clusters (eg, covered with platelets, megakaryocytes, or neutrophils). In this context, the neutrophil-lymphocyte ratio in CTC-positive patients may be used to stratify prostate cancer patients with metastatic status.^6^ CTCs have gained attention as potential prognostic and predictive biomarkers, as they provide a source of tumour-specific information and can be easily repeated throughout the treatment and disease progression.^7^^,^^8^ Liquid biopsy has been known for the last decade as a less invasive alternative to conventional tissue biopsy, resembling tumour characteristics in the blood or other fluids (eg, cerebrospinal fluid). Despite genomic profiling of tissue biopsies provide useful information about patients’ disease status, several limitations must be taken into account. First of all, the primary tumour cannot recapitulate the phenotype of metastatic lesions. Those are particularly difficult to access in case of advanced disease stage, such as mCRPC, where frequently bone metastases are developed. In this case, CTC whole-genome sequencing may be superior due to a more accessible route to obtain information about the metastatic tumour.^9^ It is believed that liquid biopsy may have an advantage over tissue biopsy due to the known heterogeneity of prostate cancer. Moreover, the snapshot of the current state of the disease obtained with needle biopsy can lead to false-negativity, as it is considered to happen in case of neuroendocrine prostate cancer diagnosis^10^ and is challenging for the extraction of information about tumour evolution and clonal selection caused by treatment. However, in contrast to tissue biopsy, CTC analyses do not allow for simultaneous direct assessment of the tumour microenvironment.^11^ Nevertheless, many studies have demonstrated the interest of CTCs in metastatic prostate cancer as a prognostic marker. However, most studies have focused on CTCs in metastatic castration‐resistant prostate cancer and very few in oligometastatic prostate cancer or early-stage prostate cancer, where some patients may present occult disseminated disease and therefore should be studied extensively. The present review will provide a brief overview of the most recent findings on CTCs in prostate cancer, with a specific focus on their use in prostate cancer treated by RT.

## A wide diversity of methods to detect, isolate, and characterize CTCs

Most of the CTCs that enter the circulation die within 24 hours, either by anoikis or immune attack.^12^ The half-life of CTCs in the bloodstream is approximately 10 minutes for CTC clusters.^13^ CTCs are rare cell events, and prior to their detection, an enrichment approach of blood samples is mandatory to improve their isolation. CTCs can be detected and isolated by various techniques for quantitative (CellSearch, RT-PCR, Epispot, microfluidics) and qualitative analysis (immunocytochemistry, FISH, scRNA-seq), determining CTC count or phenotypic and genetic characteristics, respectively.^14^ Qualitative and semi-quantitative approaches include immunocytochemistry using fluorescence-labelled antibodies directed against epithelial cell adhesion molecule (EpCAM), cytokeratins (CKs), and CD45 (to exclude leukocytes) for instance.^15^ Among quantitative methods, the detection of PSA, AR-V7, CK19, or TWIST1 mRNA in prostate cancer allows confirmation of tumour origin. Functional assays such as Epispot (Enzyme-Linked ImmunoSpot) detect live CTCs based on secretion of tumour markers like PSA or FGF2. Single-cell sequencing can also be carried out to identify gene expression patterns of individual CTCs or FISH to identify gene amplifications or translocations in CTCs.^16^^,^^17^

Among common approaches, immunoaffinity approaches are used. Particularly, EpCAM is used as a surface cell antigen for positive selection. The anti-EpCAM antibody is coupled to ferromagnetic beads in the CellSearch system, which is the most widely used and the only one approved by the US Food and Drug Administration to monitor breast, colorectal, and prostate malignancies.^18–20^ A limitation of this approach is epithelial-to-mesenchymal transition, which allows tumour cells to intravasate into the systemic circulation and then migrate to distant organs. Because CTCs lose EpCAM during this transition, they cannot be detected by EpCAM-based methods, resulting in an underestimation of CTCs. This explains why first studies evaluating CTC role in prostate cancer patients under RT failed to prove their prognostic potential. Subsequently, non-EpCAM-based isolation approaches have been proposed.^14^ Alternative approaches are physical enrichment techniques named label-free methods. For example, ISET (Isolation by Size of Epithelial Tumour Cells) is a size-based filtration system. There, CTCs are trapped on the filter due to their larger size compared to blood cells.^21^^,^^22^ Other options rely on density gradient centrifugation, such as Ficoll-based or OncoQuick approaches, separating CTCs from other blood components.^23^ Finally, the microfluidic enrichment, such as the Parsortix system, captures CTCs based on size and deformability using microfluidic channels.^24^ Parsortix enrichment step can be coupled to dielectrophoretic methods (DEPArray) to quantify and isolate pure single CTCs^25^ similarly to CellSearch. These approaches are EpCAM independent and can therefore detect and capture dedifferentiated CTCs which remained underinvestigated (Table 1).

**Table 1.** CTCs isolation methods including their clinical utility, advantages, and disadvantages.

Method	Clinical utility	Advantages	Disadvantages
CellSearch^26^^,^^27^	FDA-approved method for CTC detection in mCRPC. Allows to CTCs as surrogate marker for treatment response	Allows for whole blood analyses and is compatible with low-pass sequencing of copy number variations proving CTCs origin	Exclusive selection of EpCAM^hi^ CTCs, therefore reducing heterogeneity of detected CTCs. Necessity to fix samples restricts functional analyses. Even in metastatic disease detection rate is relatively low
RNA-based methods (eg, RT-PCR, ISH)^28–34^	Allows assessment of classical markers of PCa progression in combination with additional markers (eg, stemness and therapy resistance)	Cost-efficient, specialized equipment is not required, provide quantitative analyses	For multiplex single-cell analyses may require material amplification introducing some bias into the final readout
Epispot^35–37^	Provides similar results as serum PSA levels but ensures cancer-specificity. Have a promising value for localized PCa	Detects metabolically active viable CTCs based on the secretion of tumour markers (PSA, FGF2)	Lacks standardization, different protocols are used across different research laboratories. May miss more aggressive subpopulation of neuroendocrine or AR-neg cells downregulating PSA
Microfluidic devices (eg, Parsortix)^24^^,^^38^	Useful for isolation of cells undergoing EMT. Possibly applicable for selection of CTCs in both localized and metastatic PCa	Label-free approach may help to capture broad CTC population, including CTC clusters. Compatible with several additional assays (eg, genomic and transcriptomic analyses). Quick sample processing	Large number of leukocyte contaminant may lead to CTC false positivity
Immunocytochemistry, immunofluorescence and microscopy analyses^34^^,^^39–41^	Easy to use for multiplex markers analyses—allowing to combine classical one with novel candidates that may require additional validation	May provide with high-resolution images, allow to visualize single CTC and further morphological analyses. Useful for live and fixed analyses	Time required for enumeration might be consuming unless combined with semi-automated approaches. Multiplex staining may lead to background signal
Filtration-based methods (eg, ISET, CanPatrol)^28^^,^^42^	Due to high heterogeneity of isolated CTCs may have predictive value for non-metastatic PCa	Label-free approach, captures CTCs with mesenchymal and epithelial phenotype	Large number of contaminants, thus it needs to be combined with additional methods (eg, immunostaining). May miss smaller CTCs
Density gradient centrifugation^23^	Covers CTC heterogeneity, therefore shows utility in novel phenotypes investigation	Allows capturing heterogeneous pool of CTCs and is extremely cost efficient	Further selection is required due to contaminants, CTCs of different sizes than leukocytes may be missed
DEPArray^43^	Allow for pure single cell isolation and is a step towards personalized therapy	Allows multiparametric analyses and is compatible with functional analyses	Analytical method requiring combination with pre-enrichment steps

Abbreviations: AR = androgen receptor; CTC = circulating tumour cell; DEP = dielectrophoresis; EMT = epithelial-to-mesenchymal transition; EpCAM^hi^ = high expression of epithelial cell adhesion molecule; EPISPOT = Epithelial ImmunoSPOT; FDA = Food and Drug Administration; FGF2 = fibroblast growth factor 2; ISET = isolation by size of epithelial tumour cells; ISH = in situ hybridization; mCRPC = metastatic castration-resistant prostate cancer; PCa = prostate cancer; PSA = prostate-specific antigen; RT-PCR = reverse transcription polymerase chain reaction.

## Impact of RT on CTCs release

RT is a cornerstone of cancer treatment that uses high-energy rays or particles to destroy or damage cancer cells while minimizing harm to surrounding healthy tissue.^44^^,^^45^ It can be used as a curative treatment, either alone or in combination with surgery and chemotherapy, depending on the type and stage of cancer. In advanced cases, it is also employed palliatively to relieve symptoms such as pain, bleeding, or obstruction, thereby improving the patient’s quality of life.^46^^,^^47^

The therapeutic management of patients with radiotherapy in oncology has led to an analysis of the impact of radiation on the release of CTCs from the irradiated tumour mass into the bloodstream. In preclinical animal models of non-small cell lung cancer and squamous cell carcinoma, a transient increase in the number of CTCs has been reported immediately following RT with non-curative intent.^48^^,^^49^ This raises the question of whether the mobilization of CTCs during radiation may cause metastasis.^50^ Martin et al investigated whether radiotherapy could mobilize viable CTCs in patients with advanced locoregional or metastatic disease. Seven of 9 patients treated with palliative radiotherapy had increased CTCs 24 hours after the first fraction compared to baseline. In a further 4 out of 8 patients treated with curative RT, CTCs also increased and had elevated levels of γ-H2AX consistent with exposure to high doses of ionizing radiation. The CTCs isolated in this study were viable as they were positive for Ki67 staining and had the ability to proliferate *in vitro*.^50^ Koonce et al^49^ showed in the 4T1 syngeneic mouse model of triple-negative breast cancer that while RT induced a short-term increase in CTCs, there was a subsequent significant reduction in lung metastases. There were no differences in CTC numbers between the irradiated mice and the control mice at 8 hours, and irradiation did not correlate with an increase in metastases, suggesting that the initial mobilization of CTCs does not correlate with an increased risk of metastasis. In contrast, Vilalta et al^51^ suggested that even those irradiation-released CTCs may have increased seeding capacity. More recently, an association between locoregional failure in breast cancer after surgery and irradiation and CTCs has been established.^52^ These authors demonstrated the contribution of a subset of CTCs highly expressing ectonucleotide pyrophosphatase/phosphodiesterase 1 (ENPP1) to the local relapse by a self-seeding mechanism. This local relapse was also associated with a recruitment of polymorphonuclear myeloid-derived suppressor cells (MDSCs) and neutrophil extracellular trap formation. In this context, RT may have altered the local immune remodelling exploited by ENPP-expressing CTCs. A transient increase in CTC levels immediately after RT may also be due to tumour cell shedding caused by vascular damage.^48^^,^^49^ Post-RT alteration of the peri-tumour environment can be a consequence of blood vessels damage within tumours, further promoting the release of cells into the circulation. In addition, radiation could induce biomolecular changes in tumour cells, for example, by promoting the notorious epithelial-to-mesenchymal transition, again leading to increased CTC shedding. Photon radiation reduces endothelial cell-cell adhesion.^53^ It induces downregulation of E-cadherin and upregulation of N-cadherin, a reliable marker of the mesenchymal phenotype of cells. It increases endothelial permeability and transendothelial migration. In HUVEC cells, 2 or 4 Gy of photons increase the expression and activity of ADAM10, which in turn degrades VE-cadherin.^53^ Overall, these parameters favour the mechanism of transendothelial migration of cancer cells into the bloodstream to become CTCs.

Several prospective and observational studies have investigated CTC kinetics in the setting of radiotherapy, but the vast majority have focused on conventional photon-based treatments. Only a handful of reports have addressed particle therapy, and these typically examined surrogate biomarkers such as circulating tumour DNA (ctDNA) or immune-related parameters rather than CTCs directly. To date, no prospective study has systematically compared CTC dynamics across the major radiotherapy modalities—stereotactic body radiotherapy (SBRT), conventionally fractionated external beam RT, brachytherapy, and particle-based approaches—despite their well-recognized biological and physical differences. This represents a clear gap in the literature and an important opportunity for future research.

Emerging data suggest that SBRT, delivered in high doses per fraction, can induce an early surge of viable CTCs, sometimes in clusters, and that persistent detection after treatment may predict distant recurrence.^54^ In contrast, conventionally fractionated external beam RT is thought to release CTCs more gradually, and preclinical evidence indicates it may also promote pro-metastatic phenotypic changes such as epithelial-to-mesenchymal transition. Brachytherapy^55^ and particle therapy are hypothesized to have a more limited impact on systemic dissemination because of their highly localized or depth-specific dose distribution, leading to less irradiation of surrounding vasculature and circulating blood. Nevertheless, direct evidence is sparse. Very few studies have evaluated CTC kinetics during or after brachytherapy, and to date, no clinical trial has longitudinally assessed CTC counts or clusters in patients treated with protons or carbon ions. Instead, biomarker investigations in particle therapy have largely relied on ctDNA monitoring, immune cell dose metrics such as the effective dose to circulating immune cells (EDIC), or surrogate markers like tumour-derived exosomes.^56^^,^^57^ These indirect findings underscore the need for dedicated, modality-specific CTC studies to clarify whether different forms of radiotherapy exert distinct systemic effects relevant to metastasis.

## Biological validation of CTC enumeration in metastatic prostate cancer

Over the past decade, numerous clinical trials have demonstrated the clinical and biological value of CTC enumeration in the metastatic setting. In 276 metastatic castration resistant prostate cancer, de Bono et al^20^ reported that CTC count was a predictive marker of overall survival. The CTC count assessed by CellSearch before a new line of chemotherapy outperformed PSA in predicting overall survival. Subsequently, all reports in metastatic prostate cancer highlighted the prognostic value of CTC count at baseline^27^^,^^58^ with the CTC counts of ≥ 5 CTC per 7.5 mL determined by CellSearch being associated with a high tumour burden and poor disease outcome. An increase in CTC count during treatment has also been associated with poor overall survival.^26^^,^^58^^,^^59^

Further studies validated the clinical utility of CTC molecular features for clinical decisions. Expression of AR splice variant 7 (ARv7) status in CTCs from mCRPC patients can predict resistance to second-generation antiandrogens.^60^^,^^61^ To date, no published study has correlated CTC heterogeneity and dynamics with predictive value for radiotherapy response and metastatic progression in prostate cancer patients.

## Biological and clinical value of CTCs in localized prostate cancer

Approximately 80% of all prostate cancers are diagnosed at the local stages and their prognosis depends primarily on the clinical stage. In localized stages, the overall survival at 5 years is over 98%.^1^ However, distant metastases may occur even prior to local recurrence and are associated with a significant decrease in 5-year survival rate, down to about 30%.

RT for localized prostate cancer has become an increasingly relevant alternative for surgery, after surgery as adjuvant therapy or salvage therapy. Prognosis and treatment depend on the biopsy ISUP score, TNM score, and serum concentration of PSA. Biochemical recurrence (BCR) is defined by the American Urological Association as a detectable or rising PSA level after surgery, that is, ≥ 0.2 ng/mL.^62^ It is also defined after RT by the American Society for Radiation Oncology and Radiation Therapy Oncology Group as a rise in PSA to ≥2 ng/mL above nadir PSA.^63^ Studies have reported that BCR is associated with a significantly increased risk of metastasis and approximately 30% of patients with BCR will develop metastasis. According to a meta-analysis of salvage radiotherapy, around 58% of patients receiving radiotherapy (70 Gy) achieve relapse-free survival after 3-5 years.^64^ Thus, a large proportion of these patients experience side effects without benefit either because of local treatment failure or because of undetectable disease outside of the prostate/seminal vesicle bed at the time of treatment. Given the heterogeneous presentation of localized prostate cancer, the possibility of systemic spread that cannot always be excluded and, considering the limitations of PSA as a biomarker, such as lack of cancer specificity, it is important to identify surrogate markers stratifying patients with high risk of treatment failure who should have benefited from early use of androgen deprivation therapy (ADT).

Available data on the significance of CTCs in earlier stages of prostate cancer are sparse and mostly relate to patients who underwent prostatectomy. Detection of CTCs in localized prostate cancer using CellSearch has been reported with a lower positivity rate than in CRPC, of only 5-27%.^55^^,^^65^ Cieślikowski et al^66^ showed in a first study that patients with disseminated cancer had significantly higher CTC counts as determined by the CellSearch. Eighty-five patients with localized prostate cancer at the baseline had ≥ 1 CTCs determined by CellSearch. This was a significant predictor of worse overall survival during at least a 5-year follow-up.^67^ However, they did not find any correlation between the CTC count and the metastasis-free survival. Therefore, more than baseline CTC count, this observation should imply that CTC count should be monitored longitudinally at specific time intervals to be considered as a prognostic marker in localized prostate cancer. Recently, Schott et al investigated CTC dynamics during definitive or postoperative RT. They used an EpCAM-based method and showed that most of patients experienced a decrease in CTC numbers after RT, especially in the adjuvant situation.^68^ Patients with increasing CTCs after radiotherapy had significantly more relapses than those with decreasing CTCs. This is consistent with the observation in breast cancer, where patients with increasing epithelial CTC numbers at the end of adjuvant radiotherapy had a significantly higher risk of relapse.^69^ Nevertheless, it is essential to note that some patients with increasing CTC levels did not relapse. This highlights the need for additional markers to better define patients who may benefit from hormonal therapy.

In the BlastM prospective trial, patient blood was collected before treatment, radical prostatectomy or RT with ADT, at the end of RT, 3 and 9 months post-RT, and between 2 and 2.5 years after completion of all treatment.^70^ Epithelial and prostate-specific markers were used to identify and quantify CTCs. Patients were divided into biochemically favourable (PSA < 1 ng/mL) and unfavourable (PSA > 1 ng/mL) at 3 months after RT. In 101 patients, CTCs were enumerated in 366 blood samples over time. CTCs were undetectable in 9% at baseline, 18% at the end of RT; 33% at 3 months, 36% at 9 months, and 44% at 2-2.5 years. There was a significant trend towards a decrease in CTCs after treatment.^70^ In a separate analysis, they compared the change in CTCs in the biochemically favourable and unfavourable groups. CTC counts were more significantly reduced over time in those with a favourable early biochemical response, suggesting a potential cause-and-effect relationship between CTC persistence and outcome. There was no correlation between CTC counts and PSA pre-RT, but there was a reciprocal relationship at 3 months post-RT.^70^ In a cohort of 241 patients treated for low- or intermediate-risk prostate cancer, Murray et al^71^ found that CTC-negative patients had fewer treatment failures 3 months after RT and a significantly longer median time to BCR than CTC-positive patients, highlighting the association between tumour progression and CTC counts. Lowes et al^40^ evaluated the presence of prostate CTCs at baseline and at multiple time points (6, 12, and 24 months after treatment) in prostate cancer patients undergoing adjuvant or salvage radiotherapy after prostatectomy. They described a significant positive correlation of CTC status with positive tumour resection margins and a trend towards significance with the presence of seminal vesicle invasion and extracapsular extension, predictive of poor response to RT. They found no correlation between PSA level and CTC count. CTC-positive status at any time predicted time to biochemical failure.^40^ The small number of patients enrolled (*n* = 55) and the CTC positive rate using CellSearch was only 29% are the main limitations of the study. Gunnlaugsson et al^72^ reported a prospective trial including 61 patients with recurrent prostate cancer after prostatectomy and treated with RT. CTC count with CellSearch failed to detect early relapses after salvage radiotherapy because it was only exceptionally detectable in patients with early biochemical relapse at low PSA levels.

Thus, CTC count does not always seem to correlate with other clinicopathological parameters (PSA, TNM, Gleason). Following the dynamics of CTC number appears more informative. However, most of the mentioned studies included rather small numbers of patients and future studies with larger samples and longer follow-up time may provide new insights into the CTC-specific prognostic potential for metastatic progression after RT.

## Biological and clinical value of CTCs in metastatic prostate cancer

The natural history of prostate cancer is often associated with the development of metastatic foci related to the dissemination of CTCs. Although initially identified as a process originating from a single CTCs, polyclonal seeding and cooperation between multiple subclones is now well recognized.^73^ This interclonal cooperation results in the ability of CTCs to spread from one metastatic site to another and/or to enrich the primary tumour. In this context, the metastatic process is auto-amplified by the migration of CTCs, which strongly contribute to the development of resistance to ADT in prostate cancer.^74^ Clinically, prostate cancer is highly heterogeneous, ranging from indolent tumours to highly aggressive disease. Oligometastatic cancer is an intermediate stage between localized and widespread metastatic disease, with a low number of metastatic lesions (usually less than 5).

Patients benefiting from multimodal treatment approaches present less biologically aggressive, slow-growing tumours. They particularly benefit from local ablative treatment of visible lesions with improvements in overall survival, progression-free survival, and time to initiation of systemic therapy.^75–77^

Due to differences in prognosis, we very often identify newly diagnosed, low-burden metastatic prostate cancer, which is considered as “oligometastatic” disease, and failure after radical treatment, known as “oligorecurrent” disease. The standard-of-care for newly diagnosed low-volume metastatic disease is hormone therapy, but studies suggest that local treatment of the prostate may provide a survival benefit. Randomized controlled trials, such as the STAMPEDE trial,^78^ showed that external beam radiotherapy to the prostate added to standard-of-care (ADT ± docetaxel), improved biochemical progression-free survival and failure-free but not overall survival. However, in a subgroup analysis of patients with low metastatic burden, the trial demonstrated an improved 3-year overall survival in patients receiving RT compared to standard of care. The HORRAD trial compared ADT combined with external beam RT to hormone deprivation alone and found a nonsignificant improvement in overall survival for the external beam RT arm in the subgroup of patients (probably underpowered) with low metastatic volume.^79^ Recently, the PEACE 1 trial, which combined RT with standard-of-care plus abiraterone, demonstrated that radiation of the prostate improved radiographic progression-free survival and castration-resistance-free survival, but not overall survival in patients with low volume de novo metastatic castration-sensitive prostate cancer.^80^ In oligorecurrent disease, the treatment of choice for distant metastases is ADT due to its ability to improve survival, but with very frequent adverse effects. Metastasis-directed therapy is increasingly being studied given the nature of low-volume oligometastatic disease in these patients. SBRT is an effective metastasis‐directed therapy that provides optimal local control and has been assessed for its potential to defer ADT initiation and improve quality of life.^75^^,^^81–83^ In this regard, the European STOMP trial randomized hormone-naïve patients who had developed asymptomatic, recurrent prostate cancer following radical treatment to surveillance or metastasis-directed therapy by surgery or SBRT of all detectable lesions.^84^ A difference in 5-year ADT-free survival in favour of the metastasis-directed therapy group was reported. The ORIOLE prospective trial demonstrated the ability of metastasis-directed therapy to prolong progression-free survival over observation in metachronous oligometastatic settings, reporting few toxicities in men treated with SBRT alone (median progression-free survival not reached vs 5.8 months).^81^ Local prostate radiotherapy as metastasis-directed therapy could be beneficial in men with low metastatic burden. However, response to treatment is heterogeneous from patient to patient.^85^^,^^86^

In the clinical trials described above, metastatic staging was based on bone scan or CT. Techniques such as choline‐ or PSMA‐PET/CT, which complement PSA evaluation, improving the detection of oligometastatic prostate cancer^87^ are more commonly used today. Although it accurately identifies low-volume disease, it detects higher number of smaller metastatic lesions, and therefore mainly identifies patient that would be classified as high-volume disease. This limitation highlights the need to develop predictive biomarkers for risk stratification and tests for therapeutic benefit before the treatment. CTCs could thus be used as a minimally invasive prognostic indicator to differentiate oligometastatic prostate cancer patients with indolent disease who could be treated by exclusive SBRT alone from those with aggressive disease requiring systemic or combined approaches.

Recently the ORIOLE cohort was used to evaluate the prognostic value of CTCs in oligorecurrent hormone‐sensitive prostate cancer with 1 to 3 asymptomatic metastases.^88^ The authors found a high positive CTC rate of 92.8% at baseline and 90% at 6 months. There was no association between the presence of CTCs (AR^+^, PSMA^+^, or VIM^+^ expression) and age, baseline Gleason, pre-metastasis directed therapy PSA, site of metastasis, or initial treatment modality. This could be potentially also explained by the use of standard PCa markers.^89^ Stereotactic ablative body radiotherapy-based metastases-directed therapy significantly decreased mean PSMA^+^ CTCs at 6 months after treatment. No correlation with progression-free survival was observed between the increasing or decreasing trend of CTCs after stereotactic ablative body radiotherapy-based metastasis-directed therapy, but progression-free survival was significantly lower in the patients with AR^+^ CTCs compared to AR^−^ CTCs at 6 months in the stereotactic ablative body radiotherapy arm. In a prospective analysis of 43 oligometastatic patients with various solid tumours, including 12 prostate cancer patients, Sud et al^90^ showed that a CTC clearance to ≤ 15/mL after 130 days by the end of stereotactic ablative body radiotherapy was associated with a durable control of irradiated lesions. In a prospective study of 35 oligorecurrent hormone‐sensitive prostate cancer patients with 1‐3 nodal and/or bone metastases undergoing SBRT, CTC levels were measured at baseline, 1 month, and 3 months after SBRT.^91^ There was no significant change in CTC level after SBRT. Multivariate analysis identified high (≥ 5/7.5 mL) CTC counts at baseline (median distant progression-free survival 29.7 vs 14.0 months) and having significantly more than 1 metastasis (median distant progression-free survival 34.1 vs10.7 months) as independent predictors of distant progression‐free survival. CTC assessment successfully stratified patients with a single metastasis (median distant progression-free survival 42.1 vs 16.7 months), but not those with more than 1 metastasis. In addition, a combined score based on CTC levels and the number of metastases effectively stratified patients. The authors suggest that baseline CTC counts are indicative of tumour metastatic potential and aggressiveness but that longitudinal changes in CTC levels after SBRT are not informative.

## Heterogeneity of prostate CTCs

Relying on classical markers/global population, CTC count alone does not appear to be sufficient to predict response to RT. CellCollector-based CTC analyses allow for CTC capture directly from the blood stream. However, in patients with localized disease undergoing radiotherapy, only 40% patients were positive for epithelial CTCs panCK.^92^ On the other hand, the same CTC population is enriched in more aggressive neuroendocrine prostate cancer and worse prognoses are associated with loss of suppressor genes (RB1, TP53, PTEN).^93^ Case study report suggested that CTCs mutational landscape cannot be revealed by their classical identification.^94^ Genomic instability score of CTCs was proposed to be more informative than simple CTC enumeration or tumour bulk analyses. This better reflects to prostate cancer aggressiveness during cabazitaxel ± carboplatin treatment (NCT01505868). Among the most common alterations, genes associated with homologous recombination deficiency and metastasis were found, for example, BRCA2 loss, Myc gains.^95^ Genomic instability contributing to CTC heterogeneity results in a morphological diversity. CTCs carrying large-scale transitions chromosomal breaks leading to chromosomal gain or losses of at least 10 Mb are typically smaller, rounder, and present increased expression of CK and AR.^96^ Secondary analyses of CTCs within the PROPHECY trial (NCT02269982) showed that CTC chromosomal instability combined with CellSearch enumeration, AR-V7 status, and clinical characteristics can inform about shorter progression-free and overall survival of mCRPC treated with abiraterone and enzalutamide. Interestingly, neither CTCs’ chromosomal instability nor neuroendocrine CTC phenotype was associated with PSA changes. This can be explained either by the low detection rate of neuroendocrine and AR-V7 CTCs or by the lack of a specific association of analysed biomarkers with treatment resistance.^10^^,^^33^ AR and AR-V7 status on CTCs have been associated with therapy failure upon administration of enzalutamide and docetaxel (PRESIDE, NCT02288247, phase 3 b randomized trial).^97^ Recent evidence has shown that specific factors influence the metastatic potential of CTCs, such as clonal diversity, clustering formation, and the co-occurrence with immune cells. Bogdanova et al^98^ evaluated the predictive performance of CTC-derived RNA and plasma-derived RNA for the benefit of radiotherapy in oligometastatic prostate cancer relapses. In 44 patients, they showed that in plasma-derived RNA, 7 transcripts (*MTCO2, ELK1, IRF2, MAPK4K, NFE2L2, ATM,* and *CDC25*) were associated with disease remission after radiotherapy. However, there was no correlation between CTC-derived DNA and radiotherapy benefit. Klusa et al hypothesized that CTCs from prostate cancer patients may share features of radioresistance and have a potential to predict response to radiotherapy and/or metastasis. They showed that chemokine receptor 4 (CXCR4), which was previously shown as stemness marker, is highly expressed in radioresistant tumour subclones and dynamically regulated after irradiation in parental cell lines^99^^,^^100^ In their study, they characterized CTC phenotypes in metastatic prostate cancer patients with progressive disease at 3 different time points during radiotherapy. While the total number of CTCs decreased after radiotherapy, the authors identified a subpopulation of CTCs expressing CXCR4 whose numbers remained stable up to 3 months and correlated with biochemical response and metastatic spread. This cell type may provide a way to dynamically monitor patients during therapy and identify those who are most likely to progress.^101^ Other studies also investigated the role of stemness markers on CTCs. For instance, a specific methylation profile of stemness regulators, such as OCT4, NANOG, and SOX2, was found in CTC clusters.^102^ Dependency on hypoxia of breast CTC clusters was associated with higher metastatic capacity.^103^ Increased expression of intercellular adhesion molecule 1 (ICAM1), a molecule responsible for cluster formation, in lung metastases of patient-derived xenograft models leads to upregulation of the stemness gene aldehyde dehydrogenase (ALDH)1A1^104^ associated with a higher tumorigenic potential.^105^

Due to the described CTC heterogeneity and discrepancy between the CTC association with PSA levels, it seems that there may not exist a standard model for all types of disease stage and selected treatment. As such, instead of applying the golden standard threshold of 5 CTCs per 7.5 mL of blood, their role may be considered more categorically, either CTCs are present or not. That way has been suggested after the first cycle of chemotherapy, where CTCs can have an advantage over classical approaches, such as CT scan or PSA evaluation, which can be performed at a later time after treatment.^106^ A similar conclusion was drawn when mCRPC patients were evaluated for the EpCAM^+^Vimentin^+^ CTCs, where the cut-off also needs to be adjusted to the newly evaluated CTC phenotype.^107^ Only mesenchymal CTCs showed association with the number of detected metastases and had higher predictive power for mCRPC-free survival.^108^ Eventually, it was proposed that, to test the clinical feasibility of novel markers, exclusively, CellSearch should be used as the FDA-approved tool for CTC detection. It should be used before any further analyses, allowing characterization of single-cell CTC, for example, K19^+^ARV7^+,^ and coupling with a qPCR-based assay.^29^ A similar combinatory approach was suggested for non-metastatic patients (Phase III trial, NCT00638690) when CTC were evaluated next to lactate dehydrogenase.^109^

It can be debated, if the reliance on pre-selected markers can be fully informative about treatment selection or whether total CTC heterogeneity could have a higher value. CTC heterogeneity is often simplified and considered as a general prerequisite for therapy failure. However, their heterogeneity level can indicate patients in favour for one treatment over another (eg, ARSI vs taxanes).^110^ CTCs play a crucial role in the metastatic spread and treatment resistance of prostate cancer, particularly in oligometastatic and oligorecurrent stages, where their detection and characterization may inform prognosis and guide therapy decisions. While SBRT shows promise in managing low-burden disease, CTC count alone is insufficient to predict response, with emerging biomarkers like CTC phenotype, RNA expression, and clonal traits offering potential for better stratification.

## Impact of androgen-deprivation therapy on CTC release

Patients treated with RT often have a history of accompanying ADT^78^^,^^111^; however, its role in the CTC release modulation during RT remains an underexplored area. Nevertheless, there are several indirect pieces of evidence that this therapy can modulate CTC occurrence even before the implementation of the RT fractionation schedule. First of all, CTCs can travel in the circulation as clusters with immune cells^112^ ADT was demonstrated to affect prostate tumour microenvironment, favouring immunosuppression driven by MDSCs and regulatory T cells (Treg) cells.^113^^,^^114^ Positive association between CTC count and immune cell frequencies (NK cells, T cells, platelets) was found in advanced prostate cancer patients participating in the 6-month exercise program (ExPeCT trial, NCT02453139). Among enrolled patients, ADT was the most common treatment combined with second-generation anti-androgens, and/or chemotherapy; however, this was not an inclusion criterion, and direct association with the treatment was not evaluated.^18^^,^^115^ Variety of CTCs is also represented by distinct CTC clusters composition, for example, they can consist of 2-100 CTCs within one cluster and their periphery is mainly defined by epithelial one (pan-cytokeratin or PSA-positive) with mesenchymal or hybrid core (vimentin-positive or pan-cytokeratin and vimentin or SERPINE1 double positive).^34^ Other studies showed that SERPINE1 plays a role in cancer inflammation, exerting anti-apoptotic activity and facilitating CTCs extravasation in cooperation with neutrophils.^116^ The intermediate E/M phenotype also has an impact on the ability of CTCs to cluster with tumour-associated macrophages. Moreover, those CTCs can be described by specific mechanical fitness associated with high adhesion and softness. This strong correlation between CTCs and macrophages was particularly found in patients before ADT start, which was less pronounced post-ADT, despite CTCs’ mechanical fitness being maintained.^117^ There is no evidence that CTC count can indicate patients responding to hormone therapy such as ADT. This seems to be rather a consequence of the CellSearch EpCAM-based method detection. Analysis of cell surface vimentin on CTCs suggests that those have better prognostic potential in mCRPC than EpCAM ones.^39^ It appears that ADT primarily targets epithelial cells, as evidenced by studies in oligometastatic hormone-sensitive prostate cancer patients, where further detection of bone metastases was linked to CTCs of mesenchymal origin.^108^ This is further supported by a prospective phase II study (*NCT01800058*) which detailed the dynamics of CTCs enumeration by CellSearch during the course of RT and adjuvant hormone deprivation in high-risk prostate cancer followed for a median period of 55 months. They showed a significant association between CTC conversion after treatment (no CTCs before treatment and ≥1 CTC/7.5 mL after treatment) and advanced tumour stages (T3 and N1). However, they had a low rate of CTC detection, and only 8 out of 62 patients had detectable at a maximum of 1 CTC during ADT, which in turn ranged between 1 and 136 after RT in 11 out of 59 patients.^118^ Overall, no studies explored the role of ADT on CTC in patients undergoing RT; however, it seems that ADT administration relative to the start of radiotherapy may influence patients’ prognoses.^119^ On the other hand, androgen receptor signalling inhibitors (ARSI) such as abiraterone acetate/prednisone or enzalutamide did not affect CTC checkpoint inhibitors (PD-L1, PD-L2, B7-H3, CTLA-4) heterogeneity in mCRPC patients; instead, clear differences were observed between the castration-sensitive and resistant groups, with higher PD-L1 CTC frequencies in the latter group.^120^ Furthermore, an exploratory study demonstrated that even ARV7 alone is not CTC-specific. Still, its expression is also found in PBMCs during abiraterone, enzalutamide, or taxanes treatment. This suggests that anti-hormonal therapies may induce immunomodulation, which can influence CTC release or detection without proper marker selection.^121^

Therefore, CTC-positivity depends on the marker selection and fluctuates during the application of hormonal therapy.^122^^,^^123^ Additionally, intra-patient heterogeneity makes it challenging to interpret the obtained data.^124^ This overall shows that hormonal treatment and other AR signalling targeting therapies influence CTC enumeration. However, it has not been investigated in depth whether it reflects CTC passive or active dissemination, or whether the current detection methods limit it. The majority of studies investigating CTCs in mCRPC disease include patients treated with ADT; however, to our knowledge, no studies have considered such evaluation before the start of ADT in a prospective randomized study, which would be of interest to understand and address the actual impact of RT in those patients.

## Limitations of CTCs as a predictive tool for RT response

One barrier to widespread use of CTC enumeration in routine clinical practice is the isolation methods of CTCs. The ratio of white blood cells and CTC concentration is approximately 10^7^ white blood cells for a standard 7.5 mL blood tube, compared to sometimes 1 CTC per tube. In addition, some CTCs resemble monocytes in size and morphology and may be hidden within the leukocyte aggregates. In addition, the release of CTCs from tumours is not constant and can vary at different times within the same patient. CTCs can disappear within minutes after release into the bloodstream, so the timing of the blood collection may be crucial.^125^ Several factors have been considered to promote CTC release. Donato et al^103^ demonstrated that the intravasation of highly metastatic CTC clusters is caused by hypoxia. There are also reports suggesting that CTC release may be regulated by the circadian rhythm and accelerated during the resting phase.^126^^,^^127^

Another limitation is the considerable discrepancy in the number of cells determined with different commercially available systems. Kuske et al^35^ compared the number of CTCs in the peripheral blood of 107 patients with high-risk prostate cancer before and 3 months after radical prostatectomy. CTCs were detected with 3 commercially available systems, CellSearch, CellCollector, and Epispot. The proportion of CTC-positive patients varied from 37% for CellSearch to 54.9% for CellCollector and 58.7% for Epispot. A significant decrease in the proportion of CTC-positive patients after prostatectomy (66% vs 34%) was demonstrated only with the CellCollector system. The number of CTCs detected with Epispot correlated significantly with serum PSA and the clinical stage of prostate cancer patients. They reported a cumulative positive rate of 81.3% using the 3 methods, including 21.5% of patients with ≥5 CTC per 7.5 mL. It has been hypothesized that a combination of detection methods should be used to improve CTC enumeration and characterization. In 15 patients with early intermediate or high-risk prostate cancer, Welsch et al^128^ used the Parsortix assay combined with RT-qPCR to improve CTC detection. They reported a high positive rate of CTC-related transcripts of 66.7% at baseline and 76.9% after RT using a threshold of 1 transcript. Using a stricter threshold of 3 positive transcripts, the positivity was only 14.3% before and 50% after RT. By immunofluorescence staining, CTCs were detected in only 20% of samples before and 14% after RT.

The abscopal effect, where localized RT leads to regression of both the primary tumour and distant metastases, has been documented in various studies.^129^^,^^130^ In this way, immune cells could eliminate the primary tumour and the disseminated distal cells. On the contrary, several authors have shown that RT is sometimes responsible for an increase in the number of metastases. Different types of radiation may have different effects on metastasis. For example, some authors have shown that photon radiation may increase the number of metastases compared to particle radiation.^131^ However, studies on the interaction of CTC formation and dissemination with different types of radiation dose, energy, and dose/rate are still scarce. By evolving as tumour foci progress and during tumour progression, CTCs change their molecular characteristics and express new differentiation clusters and mutational profiles. They could partially reflect the spectrum of tumour mutations and be considered as a snapshot of the disease evolution at a real-time.^132^ CTCs could thus be genotyped and functionally characterized to study and target the evolving mutational landscape of primary and/or metastatic tumours and, in particular, radioresistance characteristics.

## Future direction of biomarkers in prostate cancer

The incorporation of CTC analysis into radiation oncology practice offers opportunities to personalize treatment beyond conventional clinical and imaging-based risk factors. Serial CTC monitoring may therefore serve as an adjunct to PSA kinetics for early identification of patients at risk of BCR following definitive RT. Additionally, molecular characterization of CTCs could provide insight into mechanisms of radioresistance, such as DNA damage repair alterations, with potential relevance for biomarker-driven radiosensitization strategies.

An emerging area of interest is whether CTC dynamics can serve as early biomarkers to guide radiation dose adaptation. Rising or persistent CTCs during RT could indicate suboptimal tumour control, warranting treatment intensification, while rapid CTC clearance might support de-escalation strategies in selected patients. Such approaches would parallel adaptive RT concepts already being explored with imaging biomarkers. Furthermore, CTC enumeration and molecular profiling may serve as surrogate endpoints in clinical trials, allowing earlier evaluation of RT efficacy before conventional clinical outcomes such as BCR or metastasis-free survival become apparent. Prospective studies are needed to validate these applications, but they highlight the potential for CTCs to support a shift towards biologically adaptive RT in prostate cancer.

Tissue-based genomic assays such as Decipher and PORTOS have been integrated into RT decision-making, particularly in guiding the use of adjuvant or salvage radiation in post-prostatectomy patients.^133^^,^^134^ While these classifiers offer static baseline prognostic information, they cannot capture tumour dynamics during or after RT. In contrast, CTCs may serve as a longitudinal biomarker, enabling adaptive RT strategies and the early detection of treatment escape.

In the BLaStM trial (NCT02307058), association between tissue-based gene signatures (including Decipher, PORTOS, PAM50) and CTC counts in intermediate/high-risk prostate cancer was assessed. Higher CTC counts correlated with PORTOS (predictive of RT response; RR 6.00, *P* < .001), PAM50_basal (predictive of hormone response RR 2.21, *P* < .001), and Decipher (RR 1.16, *P* = .048), suggesting that CTC burden may reflect underlying genomic risk and radiosensitivity profile.^135^ This supports the notion of pairing baseline genomic classifiers with dynamic CTC monitoring and particular genomic classifiers for baseline risk assessment and CTCs for real-time monitoring to refine patient selection for treatment intensification or de-escalation.

PSMA PET has significantly enhanced recurrence detection following RT and informs salvage treatment strategies. Parallel advances in liquid biopsy highlight the potential synergy between imaging and circulating biomarkers. A notable example is the analysis of the STOMP-like and ORIOLE trial cohorts, in which circulating PSMA-positive extracellular vesicles (PSMA^+^ EVs) were shown to be powerful prognostic and predictive biomarkers in oligorecurrent prostate cancer managed with stereotactic ablative radiotherapy (SABR).^136^ Low baseline PSMA^+^ EV levels correlated with improved biochemical and radiographic progression-free survival and predicted benefit from SABR. These results emphasize how liquid biomarkers may complement imaging to refine patient selection and treatment response evaluation. Although similar clinical data for CTCs are not yet available, it is plausible that CTC profiling—especially of PSMA expression—could eventually be integrated with PSMA PET and EV analyses to provide a more complete picture of disease biology before and after RT.

The incorporation of CTC analysis into radiation oncology practice offers opportunities to personalize treatment beyond conventional clinical and imaging-based risk factors. Serial CTC monitoring may therefore serve as an adjunct to PSA kinetics. Rising or persistent CTCs during definitive RT could indicate suboptimal tumour control, warranting treatment intensification. Rapid CTC clearance during treatment might support de-escalation strategies, while CTC analysis after RT could identify early risk of BCR. In oligometastatic prostate cancer, CTC count before treatment could help identify patient who would benefit the most of RT or those requiring additional treatment. Molecular characterization of CTCs could provide insight into mechanisms of radioresistance, such as DNA damage repair alterations, with potential relevance for biomarker-driven radiosensitization strategies. Such approaches would parallel adaptive RT concepts already being explored with imaging biomarkers. Prospective studies are needed to validate these applications, but they highlight the potential for CTCs to support a shift towards biologically adaptive RT in prostate cancer.

## Conclusion

Despite its promise, the use of CTC in predicting RT response in localized prostate cancer faces several challenges. Direct evidence is currently limited, because most studies enrolled a limited number of patients and determining the prognostic value of the CTC count in patients with localized prostate cancer would optimally require longitudinal monitoring of this parameter, ideally in a prospective randomized controlled trial. The prognostic value of the CTC count has been clearly demonstrated in metastatic prostate, but its utility to predict the benefit of RT in oligometastatic prostate is unclear. It seems that baseline CTC count could stratify patients who could benefit from RT due to low tumour burden. However, a decrease in CTC count after RT is not always predictive of treatment response. Advances in single-cell DNA sequencing and RNA sequencing technologies have made CTCs an important cell source for genomic profiling. Determining the molecular profiles of CTCs and/or tumours could help determine the susceptibility of an individual’s disease to radiotherapy and a kinetic genomic profiling (eg, CNV/CNA) would help to follow the clonal evolution of the disease and consequently any biological evolution. In any case, additional studies with more patients and different radiation doses are needed to draw conclusions about CTC release, metastasis, and overall survival. Ongoing research aims to address these challenges, with multidisciplinary teams using artificial intelligence to recognize CTCs and identify biomarkers that can predict disease progression and treatment efficacy.
